# Safe endoscopic removal of ingested fish bone with esophageal perforation and impingement on the aortic wall

**DOI:** 10.1002/jgh3.12701

**Published:** 2021-12-27

**Authors:** Van Trung Hoang, Hoang Anh Thi Van, Vichit Chansomphou, Uyen Giao Vo

**Affiliations:** ^1^ Department of Radiology Thien Hanh Hospital Buon Ma Thuot Vietnam; ^2^ Department of Radiology Savannakhet Medical‐Diagnostic Center Kaysone Phomvihane Laos; ^3^ Department of Vascular Surgery Fiona Stanley Hospital Murdoch Western Australia Australia

**Keywords:** computed tomography, endoscopy, esophagus, gastroenterology, upper GI

## Abstract

Esophageal foreign bodies are common conditions that may lead to serious complications, such as esophageal perforation, neck abscess, mediastinitis, arterial injury, and lung damage. We report a rare case of esophageal fish bone impingement on the aorta that was managed without complication by endoscopic removal.

## Graphical abstract

Esophageal foreign bodies are common conditions that may lead to serious complications, such as esophageal perforation, neck abscess, mediastinitis, arterial injury, and lung damage. Esophageal foreign bodies are usually managed with surgical removal. We report a rare case of esophageal fish bone impingement on the aorta that was managed safely and uncomplicatedly by endoscopic removal.

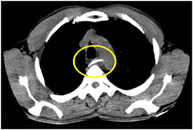

## Introduction

Esophageal foreign bodies are a common problem for gastroenterologists and surgeons. If the foreign body impinges on the aorta but does not cause serious complications, endoscopic removal of the foreign body may be considered.^1^ Here, we report a case of aortic impingement due to esophageal fish bone that was managed endoscopically without complications.

## Case report

**Figure 1:**
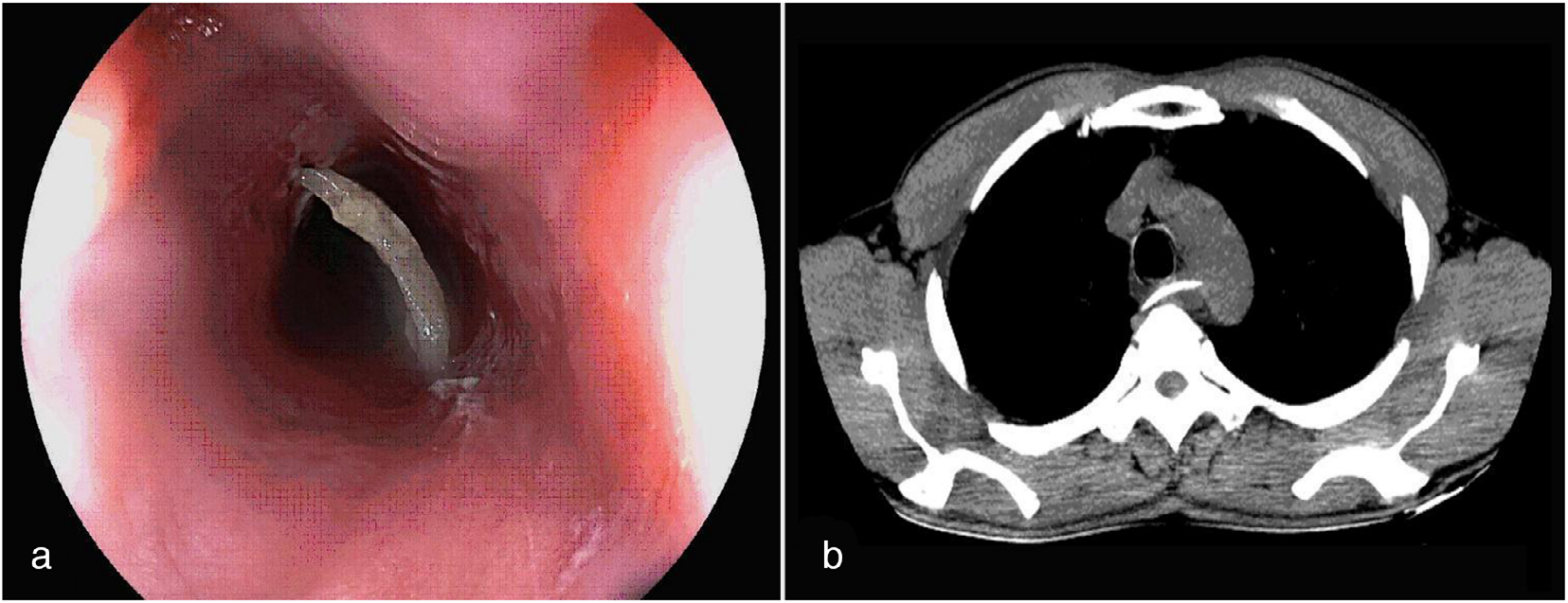
(a) The endoscopic image shows a fish bone foreign body with two ends of a fish bone plugged into the esophageal wall causing inflammation of the mucosa. (b) Chest computed tomography image shows a fish bone foreign body inside the esophagus with one end impinging on the wall of the aorta without perforation; in addition, inflammation with exudative changes around the esophagus.

A 50‐year‐old man was admitted to the hospital with persistent dysphagia after lunch. Endoscopy showed that the upper esophagus had a deep transmural fish bone foreign body and the mucosa was highly edematous, so a deep perforation was suspected (Fig. 1a); the intervention to retrieve the bone was delayed and a computed tomography (CT) scan was performed for further evaluation. CT images showed a 33‐mm linear, radiopaque foreign body inside the esophagus with one end perforating the esophagus and impinging on the aortic wall without aortic perforation (Fig. 1b). CT images showed no air in the mediastinum. Prophylactic supportive interventions in the event of potential complications were discussed prior to making an attempt at endoscopic bone removal. At a second endoscopy, the bone was successfully removed with a noose. The defect was minor and there was no bleeding: an esophagus clip was not required. Subsequently, he was discharged home on antibiotics. Endoscopy and CT scan repeated 7 days later showed a normal esophageal mucosa, and he remained well on review after 1 month.

## Discussion

Esophageal sharp and pointed foreign bodies can be life‐threatening with several complications and significant mortality. Flexible endoscopy (first‐line option, usually does not require general anesthesia) and rigid endoscopy (suitable for sharp‐pointed or large objects, usually requires general anesthesia) are the most widely used technique options for the removal of esophageal foreign bodies. Surgery is the final option when endoscopic failure or serious complications are present. Esophageal defect management includes conservation, direct surgical repair, esophageal tract reconstruction, or partial resection that depends on the characteristics of the foreign body, the location of the perforation, and the severity of the mediastinitis.^1^ In the patient described above, endoscopic removal of the fish bone was performed endoscopically and without complication.

### Patient consent

Informed consent was obtained from the patient to publish these images.
